# Interdisciplinary integration in public health emergency education: learning outcomes following a curriculum reform for preventive medicine undergraduates

**DOI:** 10.3389/fpubh.2026.1852123

**Published:** 2026-05-25

**Authors:** Jingxia Wei, Cheng Kang, Xiangyuan Yu, Yan Sun

**Affiliations:** 1 School of Public Health, Guilin Medical University, Guilin, Guangxi, China; 2 Academic Affairs Office, Guilin Medical University, Guilin, Guangxi, China

**Keywords:** curriculum reform, emergency preparedness knowledge, interdisciplinary training, preventive medicine, public health education, Shapley additive explanations

## Abstract

**Background:**

Developing a public health workforce with strong emergency response capacity is an important goal of medical education reform. However, evidence remains limited regarding how interdisciplinary and practice-oriented curricular reforms influence emergency preparedness-related knowledge acquisition among preventive medicine undergraduates. This study evaluated the effects of a curriculum reform based on an interdisciplinary, integrative, and collaborative training model.

**Methods:**

A cross-sectional study was conducted among 400 fourth-year undergraduate students majoring in preventive medicine at a medical university in China, including the 2017, 2018, 2020, and 2021 cohorts, with 100 students from each cohort. The 2017 and 2018 cohorts were classified as the pre-reform group, and the 2020 and 2021 cohorts as the post-reform group. Data were collected using an anonymous structured questionnaire covering academic characteristics, self-perceived emergency response competence, objective knowledge performance, and satisfaction with educational resources. Group comparisons, multivariable linear regression, and machine learning analyses were performed. Random Forest, Decision Tree, XGBoost, and LightGBM models were compared, and SHAP was used to interpret the best-performing model.

**Results:**

Compared with the pre-reform group, the post-reform group reported significantly greater participation in interdisciplinary courses, virtual simulation training, emergency drills, research projects, internship experience, and dual-mentor guidance. They also showed higher competence scores (36.05 ± 9.46 vs. 31.68 ± 9.89), higher satisfaction scores (21.52 ± 6.06 vs. 19.32 ± 6.41), and better objective knowledge performance (6.76 ± 1.50 vs. 5.11 ± 1.78), with all differences reaching statistical significance. In multivariable regression, interdisciplinary courses (β = 0.800, *p* = 0.002), VR simulation frequency (β = 0.446, *p* = 0.008), research projects (β = −0.382, *p* = 0.037), and internship experience (β = 0.399, *p* = 0.023) were significantly associated with objective knowledge performance. Among the machine learning models, Decision Tree showed the best overall predictive performance in both the test set (AUC = 0.713).

**Conclusion:**

An interdisciplinary and practice-oriented curriculum reform was associated with improved emergency preparedness-related knowledge acquisition among preventive medicine undergraduates. Interdisciplinary coursework, virtual simulation, and internship experience appeared to be important factors associated with better performance. These findings support the value of integrated training strategies in public health emergency education and may inform future curriculum optimization.

## 1 Introduction

Public health systems are increasingly challenged by population aging, the rising burden of chronic diseases, environmental health threats, and emerging infectious diseases (1). These complex and interconnected challenges require a workforce that is not only grounded in core public health knowledge, but also capable of responding effectively to emergencies and other rapidly evolving health risks. As a result, strengthening the emergency preparedness capacity of future public health professionals has become an important goal of medical education reform (2).

In China, public health education has entered a new stage of development under national strategies such as the Healthy China 2030 initiative and the broader reform agenda of the “New Medical Sciences” (3, 4) Within this context, medical education is expected to move beyond traditional discipline-based teaching and to promote closer integration across medicine, public health, information technology, and the social sciences (5). For public health training, this shift is particularly important because emergency preparedness depends not only on theoretical knowledge, but also on practical skills, interdisciplinary collaboration, risk assessment, communication, and decision-making in complex situations (2, 6).

However, existing training models for public health students still face several limitations. Traditional curricula often emphasize classroom-based knowledge acquisition while providing insufficient opportunities for scenario-based learning, interdisciplinary exposure, and practice-oriented training (7). This gap may weaken students' ability to apply what they have learned in real or simulated emergency contexts. In recent years, educational strategies such as interdisciplinary courses, simulation-based teaching, emergency drills, internships, and blended learning have been increasingly adopted to address these limitations (8, 9). These approaches are intended to help students develop more comprehensive competencies and improve their readiness for public health emergency response (2).

Among these strategies, simulation and technology-enhanced teaching have attracted growing attention. Virtual simulation can recreate complex public health scenarios in a safe and controllable environment, allowing students to practice decision-making, communication, and emergency response skills (10). At the same time, interdisciplinary coursework and field-based training may help bridge the gap between theoretical instruction and practical application (11, 12). Although these approaches are widely discussed in medical and public health education, evidence remains limited regarding their combined effects within a structured curriculum reform for preventive medicine undergraduates, particularly when multiple dimensions of learning outcomes are assessed simultaneously.

Another challenge in educational evaluation is how to capture the effects of curriculum reform in a more comprehensive and interpretable way. Conventional analyses can identify associations between training experiences and student outcomes, but they may not fully characterize the relative contribution of different educational components. With the increasing availability of data-driven approaches, machine learning offers a supplementary tool for evaluating educational interventions and exploring factors associated with student performance. When combined with interpretable methods such as SHAP, these approaches may provide additional insight into which elements of curriculum reform are most closely related to learning outcomes (13–15).

Against this background, our institution implemented a curriculum reform based on an interdisciplinary, integrative, and collaborative training model for preventive medicine undergraduates. The reform emphasized interdisciplinary courses, virtual simulation training, emergency drills, research participation, internship experience, and dual-mentor guidance. The present study aimed to evaluate the association between this reform and students' emergency preparedness-related knowledge acquisition by comparing pre-reform and post-reform cohorts. Specifically, we assessed differences in self-perceived competence, satisfaction with educational resources, and objective knowledge performance, and further explored factors associated with knowledge performance using multivariable regression and machine learning methods.

## 2 Methods

### 2.1 Study design and participants

This study employed a comparative cross-sectional design with nonequivalent groups (a quasi-experimental approach), comparing pre- and post-curriculum reform cohorts of undergraduate students majoring in preventive medicine at a medical university in China.

The curriculum reform was officially implemented starting with the 2020 cohort. Based on the timing of curriculum implementation, students from the 2017 and 2018 cohorts were classified as the pre-reform group, and students from the 2020 and 2021 cohorts as the post-reform group. The 2019 cohort experienced a transitional hybrid curriculum and was excluded to ensure a clear contrast between the two curricula. Because group assignment was determined by cohort year rather than randomization, this constitutes a nonequivalent group design.

Participants were surveyed at the same relative stage in their academic progression—in the first semester of the fifth (final) year—after all had completed the relevant core curriculum requirements. Regarding the total number of eligible students per cohort: The 2017, 2018, 2020, and 2021 cohorts each had approximately 110–120 enrolled students in the preventive medicine major at the time of the survey. From each cohort, we used simple random sampling to select 100 students. The sampling fractions ranged from 78 to 82%, which are relatively high and support the representativeness of the selected samples. Eligible participants had completed the core curriculum requirements at the time of the survey. Exclusion criteria included: (1) incomplete questionnaire responses; (2) logically inconsistent: defined as contradictory responses to reverse-coded items within the same scale, failure to pass embedded attention-check questions, or selection of mutually exclusive options (e.g., reporting “never” participating in virtual simulation training while also reporting a frequency of ≥3 times); and (3) invalid answers, and duplicate submissions. All 400 randomly selected students completed the questionnaire validly, yielding a 100% effective response rate with no exclusions. This high completion rate is attributable to the fact that the objective knowledge items contributed to students' usual course performance scores, motivating full and attentive participation.

Ethical approval and informed consent procedures are described in the Ethics approval and consent to participate section.

### 2.2 Curriculum reform

**Figure 1:**
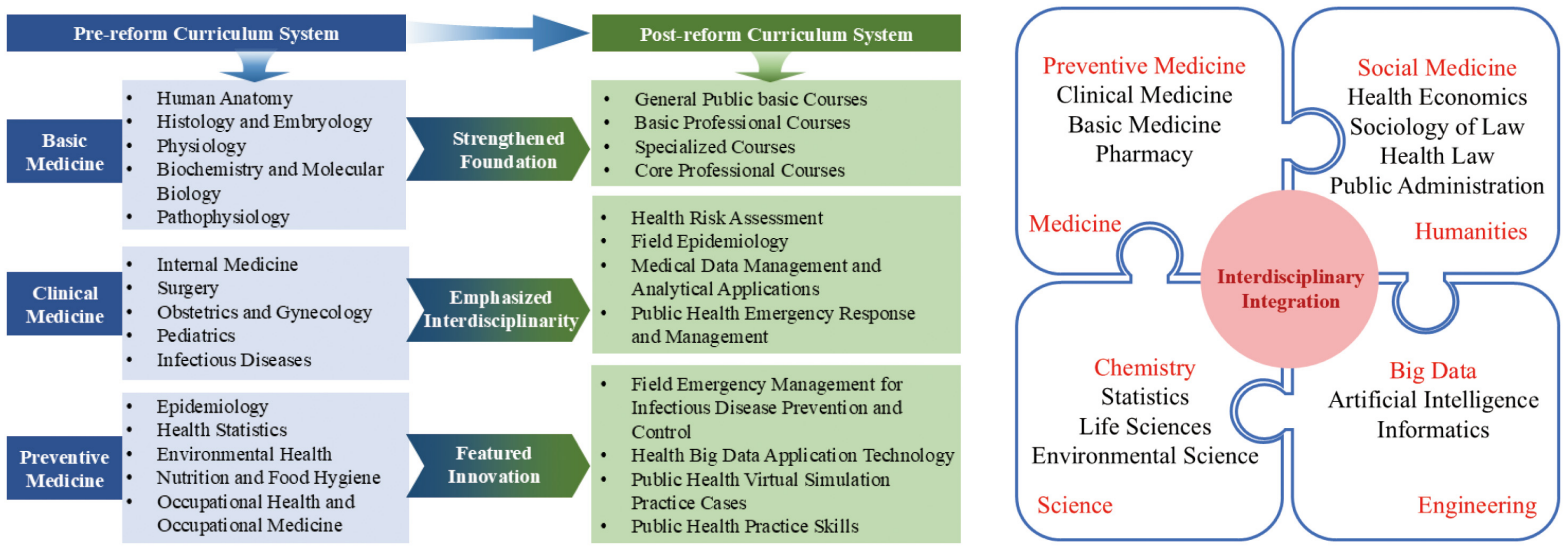
Comparison of the curriculum system before and after the reform.

The curriculum reform was officially implemented starting with the 2020 incoming cohort and was fully applied throughout their 5-year undergraduate program. The reform was delivered uniformly to all students in the 2020 and 2021 cohorts, with no individual-level variation in timing or content. The 2017 and 2018 cohorts completed the entire pre-reform curriculum without exposure to any reformed components. The 2019 cohort received a transitional hybrid curriculum and was excluded from this study to ensure a clear contrast between the two distinct curricula. Figure 1 and Table 1 provide a side-by-side comparison of key features between the pre-reform and post-reform curricula.

**Table 1:** Comparison of the curriculum system before and after the reform.

Component	Pre-reform (2017, 2018 cohorts)	Post-reform curriculum (2020, 2021 cohorts)		
		Specific content	Mode of delivery	Measurement method
Interdisciplinary courses				
	Medicine-Humanities	Medicine-Humanities; Medicine- Engineering; Medicine- Science	The teaching is conducted in the traditional classroom format, with 20% of the course sessions adopting the flipped classroom and case-based group discussion methods	dichotomous (yes/no) based on completion of all courses
VR simulation training				
	None	Emergency response for infectious disease prevention and control, Public health emergency response for natural disasters, Emergency response for environmental health incidents, and others	The implementation method is as follows: Conduct simple face-to-face reports and summaries before and after the simulation; Execute scenario drills on the national virtual simulation platform based on the network (this platform can be used on campus)	Ordinal (never = 0 times; occasionally = 1–2 times; frequently = ≥3 times) based on self-reported counts from platform logs
Emergency drills				
	Conduct a training session in the fourth academic year	Once a semester, a field-based drill is arranged, involving members from multiple disciplines. The effectiveness of the drill is further enhanced through skill competitions	The tabletop drill took place in a standard conference room with facilitators guiding discussion. The functional and full-scale drills were held at designated field training sites (an outdoor emergency simulation field and a mock command center), involving role-playing actors, simulated victims, and actual communication equipment	Total number of drill sessions attended (0–5)
Research participation				
	Optional	All students are required; To be guided by teachers and practice mentors	Project-based learning using the Gongcheng natural population cohort. Includes 12 weeks of fieldwork (in-person cohort data collection) plus online data analysis. Supervised by faculty and practice mentors	The questionnaire asks whether one has systematically participated in any research projects during their university years? (Including but not limited to innovation projects, mentor research topics, population cohort studies, publication of scientific papers, etc.)
Internship experience				
	Interned at the local CDC for 16 weeks	Interned at the local CDC for 16 weeks; Based on the empirical study of the natural population cohort in Gongcheng for 12 weeks	Outbreak surveillance, field investigation, and data reporting	Follow the teacher's evaluation; or participate in the preparation of the investigation report, or take part in the writing of the teaching case
Dual-mentor guidance				
	One academic mentor (faculty member)	One academic mentor (faculty member) and one practice mentor (senior CDC officer)	Academic mentor: Conducts theoretical teaching within the institution; practice mentor: Teaching based on real cases	Students' classroom interactions, after-class assignments, etc.

The curriculum reform was developed based on an interdisciplinary, integrative, and collaborative training model for public health emergency education. Compared with the pre-reform curriculum, the reformed curriculum placed greater emphasis on interdisciplinary courses, virtual simulation training, emergency drills, research participation, internship experience, and dual-mentor guidance. These components were intended to strengthen students' emergency preparedness-related knowledge learning outcomes and support the development of applied public health competencies.

### 2.3 Data collection and measures

Data were collected using an anonymous structured questionnaire. The questionnaire included four domains: (1) academic characteristics, (2) self-perceived emergency response competence, (3) objective knowledge performance, and (4) satisfaction with educational resources. Academic variables included academic performance, student leadership experience, participation in interdisciplinary courses, frequency of virtual simulation training, emergency drills, research project participation, internship experience, and dual-mentor guidance. The survey was administered separately to each cohort at the same relative stage of academic progression—the first semester of the fifth undergraduate year—after all participants had completed the relevant core curriculum requirements. Thus, the timing of data collection was standardized relative to each student's training pathway, although the calendar years of administration differed across cohorts.

This study assessed three types of outcomes. First, self-perceived emergency response competence was measured using a 10-item, 5-point Likert scale (1 = strongly disagree to 5 = strongly agree; total score range 10–50). The items evaluated students' perceived abilities in emergency information recognition, team collaboration, problem-solving, interdisciplinary integration, decision-making under pressure, active learning, skill application, resource integration, humanistic practice, and overall preparedness. Second, objective knowledge performance was assessed using 10 curriculum-based items: five single-answer multiple-choice questions, four true/false questions, and one sequencing question (requiring correct ordering of four emergency response steps). The total number of correct answers was calculated for each participant (range 0–10). The knowledge test was developed based on the core learning objectives of the preventive medicine program and was reviewed by three senior faculty members for content validity. Third, satisfaction with educational resources was assessed using a 6-item, 5-point Likert scale (1 = very dissatisfied to 5 = very satisfied; total score range 6–30), covering satisfaction with interdisciplinary courses, teaching case banks, virtual simulation platform, training bases, online resources, and evaluation systems.

### 2.4 Reliability and validity

The reliability and validity of the competence and satisfaction scales were assessed before formal analyses. The emergency response competence scale showed a Cronbach's α coefficient of 0.838, whereas the satisfaction scale showed a Cronbach's α coefficient of 0.760, indicating acceptable internal consistency. The Kaiser-Meyer-Olkin values were 0.920 and 0.826, respectively, and Bartlett's test of sphericity was statistically significant (*p* < 0.001), indicating that the data were suitable for factor analysis.

### 2.5 Statistical analysis

Statistical analyses were performed using Python 3.11 and IBM SPSS Statistics 27. Continuous variables were expressed as mean ± standard deviation (Mean ± SD), and categorical variables were summarized as frequencies and percentages. Categorical variables were compared using chi-square tests or Fisher's exact test as appropriate. For continuous variables, group comparisons were performed using the Mann-Whitney U test or Kruskal-Wallis test. We conducted a normality test on competence and satisfaction. The normality test yielded a *p*-value less than 0.05, but the skewness and kurtosis were still acceptable, and the parameter test was robust under *N* = 400. We performed a sensitivity analysis using non-parametric Mann–Whitney U tests and the results were consistent. Therefore, we retained the *t*-test results for the analysis of continuous variables in the main text.

To provide an overview of the strength of association between educational factors and the main outcomes, effect sizes were visualized in a heatmap. For binary variables, the absolute value of Cliff's delta was calculated. For variables with three or more categories, effect size was estimated using eta-squared (η^2^) derived from the Kruskal-Wallis test. The outcomes included competence, satisfaction, and the number of correct answers.

To identify factors associated with objective knowledge performance, multivariable linear regression analysis was conducted with the number of correct answers as the dependent variable. Independent variables included academic performance, student leadership experience, participation in interdisciplinary courses, frequency of virtual simulation training, emergency drills, research project participation, internship experience, dual-mentor guidance. Regression coefficients, standard errors, t values, and *P* values were reported. A two-sided *p* value of less than 0.05 was considered statistically significant. To adjust for potential confounding, all variables listed in Table 2 (except the outcome measures themselves) were entered simultaneously into the linear regression model. Self-perceived competence and satisfaction were not included as covariates because they were conceptualized as parallel outcome measures alongside knowledge performance, and their inclusion would introduce conceptual overlap and potential common method bias.

**Table 2:** Comparison of student characteristics before and after the reform.

Characteristic	Total	Pre-reform group	Post-reform group	Statistic	*p*	Effect sizes	95% CI
Academic performance				2.851	0.583	0.084	
< 60	25 (6.25%)	10 (5.00%)	15 (7.50%)				
60-−69	64 (12.50%)	37 (18.50%)	27 (13.50%)				
70-−79	128 (16.00%)	61 (30.50%)	67 (33.50%)				
80-−89	133 (32.00%)	67 (33.50%)	66 (33.00%)				
≥90	50 (33.25%)	25 (12.50%)	25 (12.50%)				
Student leader				0.633	0.426	0.040	
Yes	105 (26.30%)	49 (24.50%)	56 (28.00%)				
No	295 (73.80%)	151 (75.50%)	144 (72.00%)				
Interdisciplinary courses				280.851	**< 0.01**	0.838	
Yes	235 (58.75%)	35 (17.50%)	200 (100.00%)				
No	165 (41.25%)	165 (82.50%)	0 (0.00%)				
VR simulation frequency				400.000	**< 0.01**	1.000	
Never	200 (50.00%)	200 (100.00%)	0 (0.00%)				
Occasionally	116 (29.00%)	0 (0.00%)	116 (58.00%)				
Frequently	84 (21.00%)	0 (0.00%)	84 (42.00%)				
Emergency drills				179.538	**< 0.01**	0.670	
0	62 (15.50%)	62 (15.50%)	0 (0.00%)				
1	108 (27.00%)	67 (16.80%)	41 (10.30%)				
2	122 (30.50%)	71 (17.80%)	51 (12.80%)				
3	30 (7.50%)	0 (0.00%)	30 (7.50%)				
4	31 (7.80%)	0 (0.00%)	31 (7.80%)				
5	47 (11.80%)	0 (0.00%)	47 (11.80%)				
Research projects				32.724	**< 0.01**	0.286	
Yes	134 (33.50%)	40 (20.00%)	94 (47.00%)				
No	266 (66.50%)	160 (80.00%)	106 (53.00%)				
Internship experience				13.752	< 0.01	0.185	
Yes	152 (38.00%)	58 (29.00%)	94 (47.00%)				
No	248 (62.00%)	142 (71.00%)	106 (53.00%)				
Dual mentor				44.413	**< 0.01**	0.333	
Yes	107 (26.75%)	24 (12.00%)	83 (41.50%)				
No	293 (73.25%)	176 (88.00%)	117 (58.50%)				
Competence	33.87 ± 9.91	31.68 ± 9.89	36.05 ± 9.46	24,968.000	**< 0.01**	−0.451	(−0.649, −0.252)
Satisfaction	20.43 ± 6.32	19.32 ± 6.41	21.52 ± 6.06	23,926.000	**< 0.01**	−0.353	(−0.550, −0.155)
Correct answers	5.94 ± 1.84	5.11 ± 1.78	6.76 ± 1.50	30,445.500	**< 0.01**	−1.008	(−1.216, −0.800)

Bold values indicate statistical significance (*p* < 0.05).

### 2.6 Machine learning analysis

As a supplementary sensitivity analysis, machine learning methods were applied to explore factors associated with objective knowledge performance from a predictive modeling perspective, complementing the conventional multivariable regression. The goal was to assess whether the variables identified as statistically significant in the linear model were also the most predictive features in a non-linear, interaction-allowing framework. The input features included all educational and demographic variables: interdisciplinary course participation, VR simulation frequency, emergency drills, research project participation, internship experience, dual-mentor guidance, academic performance, student leadership experience. The output was a binary classification of knowledge performance, obtained by dichotomizing the number of correct answers at the median. The dataset was randomly divided into a training set (70%) and a test set (30%). Four supervised learning algorithms were developed and compared: Random Forest, Decision Tree, XGBoost, and LightGBM. Cross-validation and hyperparameter tuning were applied to the training set, whereas the test set was used exclusively for evaluating model performance. Performance metrics included area under the receiver operating characteristic curve, accuracy, sensitivity, specificity, positive predictive value, negative predictive value, and F1 score.

Among the four models, Decision Tree showed the best overall performance and was therefore selected for model interpretation. SHAP (SHapley Additive exPlanations) was used to interpret the selected model at both the global and local levels. Global interpretation included a SHAP summary plot, feature importance bar plot, heatmap, and decision plot. Local interpretation included a SHAP waterfall plot and an individual feature contribution bar plot for a representative sample. All machine learning analyses were conducted in Python using scikit-learn, xgboost, lightgbm, shap, pandas, and matplotlib.

## 3 Results

### 3.1 Comparison of student characteristics before and after the reform

A total of 400 valid questionnaires were included in the analysis. Compared with the pre-reform group, the post-reform group showed significantly greater exposure to interdisciplinary courses, VR simulation training, emergency drills, research projects, internship experience, and dual-mentor guidance (all *p* < 0.01). No significant differences were observed between the two groups in academic performance, or student leadership experience.

In addition, students in the post-reform group demonstrated higher scores for self-perceived emergency response competence (36.05 ± 9.46 vs. 31.68 ± 9.89), satisfaction with educational resources (21.52 ± 6.06 vs. 19.32 ± 6.41), and objective knowledge performance measured by the number of correct answers (6.76 ± 1.50 vs. 5.11 ± 1.78), with all differences reaching statistical significance (*p* < 0.01) (Table 2).

### 3.2 Associations between educational factors and main outcomes

**Figure 2:**
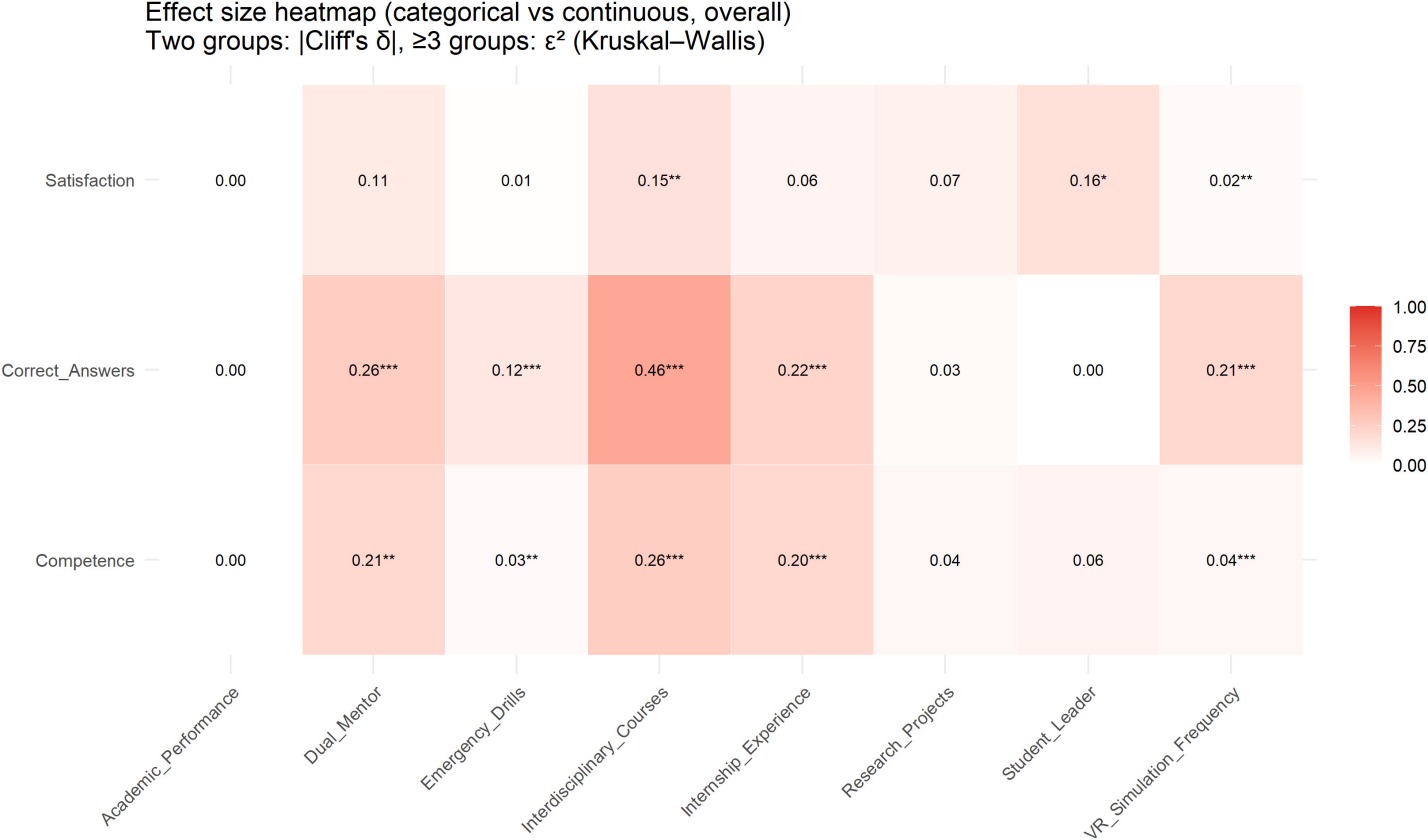
Heatmap of effect sizes for the associations between educational factors and the main outcomes. For binary variables, the absolute value of Cliff's delta is shown; for variables with three or more categories, effect size is represented by eta-squared (η^2^) derived from the Kruskal–Wallis test. Darker colors indicate stronger associations.

Figure 2 shows the strength of association between educational factors and the three main outcomes: competence, satisfaction, and the number of correct answers. Overall, competence and satisfaction showed a positive association. The number of correct answers was more strongly associated with gender, interdisciplinary course participation, frequency of virtual simulation training, emergency drills, and internship experience than with the other educational factors included in the analysis.

### 3.3 Multivariable analysis of objective knowledge performance

A multivariable linear regression model was constructed using the number of correct answers as the dependent variable. As shown in Table 3, interdisciplinary course participation (β = 0.800, *p* = 0.002), VR simulation training (β = 0.446, *p* = 0.008), and internship experience (β = 0.399, *p* = 0.023) were positively associated with objective knowledge performance. In contrast, research project participation was negatively associated with the number of correct answers (β = −0.382, *p* = 0.037). Academic performance, student leadership experience, emergency drills, and dual-mentor guidance were not significantly associated with objective knowledge performance (all *p* > 0.05).

**Table 3:** Multivariable linear regression analysis of objective knowledge performance.

Variable	Regression coefficient (β)	Standard error	t	*p*	95% CI
Const	4.001	0.378	10.587	< 0.001	(3.258, 4.744)
Academic performance	0.083	0.086	0.966	0.335	(−0.086, 0.252)
Student leader	−0.186	0.189	−0.987	0.324	(−0.557, 0.184)
Interdisciplinary courses	0.800	0.255	3.138	**0.002**	(0.298, 1.301)
VR Simulation frequency	0.446	0.168	2.653	**0.008**	(0.115, 0.777)
Emergency drills	0.421	0.248	1.700	0.090	(−0.065, 0.907)
Research projects	−0.382	0.183	−2.088	**0.037**	(−0.741, −0.022)
Internship experience	0.399	0.174	2.286	**0.023**	(0.055, 0.741)
Dual mentor	0.339	0.197	1.717	0.087	(−0.049, 0.726)

Bold values indicate statistical significance (*p* < 0.05).

### 3.4 Comparison of machine learning models

**Figure 3:**
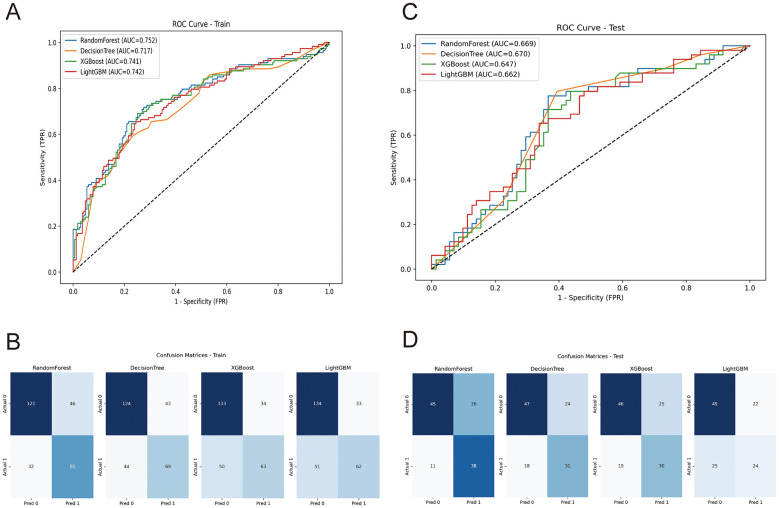
Performance of the four machine learning models in the training and test sets. **(A)** Receiver operating characteristic (ROC) curves in the training set. **(B)** Confusion matrices in the training set. **(C)** Receiver operating characteristic (ROC) curves in the test set. **(D)** Confusion matrices in the test set.

Four machine learning models were developed and compared, including Random Forest (RF), Decision Tree (DT), XGBoost (XGB), and LightGBM (LGBM). In the training set, LGBM achieved the highest AUC (0.743), followed by RF (0.735), DT (0.722), and XGB (0.710) (Table 4; Figures 3A, B).

**Table 4:** Performance of the machine learning models in the training set.

Models	AUC	AC	SE	SP	PPV	NPV	F1
Random forest	0.735	0.704	0.717	0.695	0.614	0.784	0.661
Decision tree	0.722	0.693	0.637	0.731	0.615	0.748	0.626
XG boost	0.710	0.686	0.664	0.701	0.600	0.755	0.630
Light GBM	0.743	0.711	0.593	0.790	0.657	0.742	0.623

In the test set, DT showed the best overall performance, with the AUC (0.713) and the highest F1 score (0.672). DT also achieved the highest sensitivity (0.796). The performance of RF, XGB, and LGBM was generally lower than that of DT in the test set (Table 5; Figures 3C, D). Based on these results, DT was selected as the final model for subsequent interpretation.

**Table 5:** Performance of the machine learning models in the test set.

Models	AUC	AC	SE	SP	PPV	NPV	F1
Random forest	0.678	0.675	0.776	0.606	0.576	0.796	0.661
Decision tree	0.713	0.683	0.796	0.606	0.582	0.811	0.672
XG Boost	0.680	0.658	0.633	0.676	0.574	0.727	0.602
Light GBM	0.680	0.667	0.571	0.732	0.596	0.712	0.583

### 3.5 SHAP-based model interpretation

**Figure 4:**
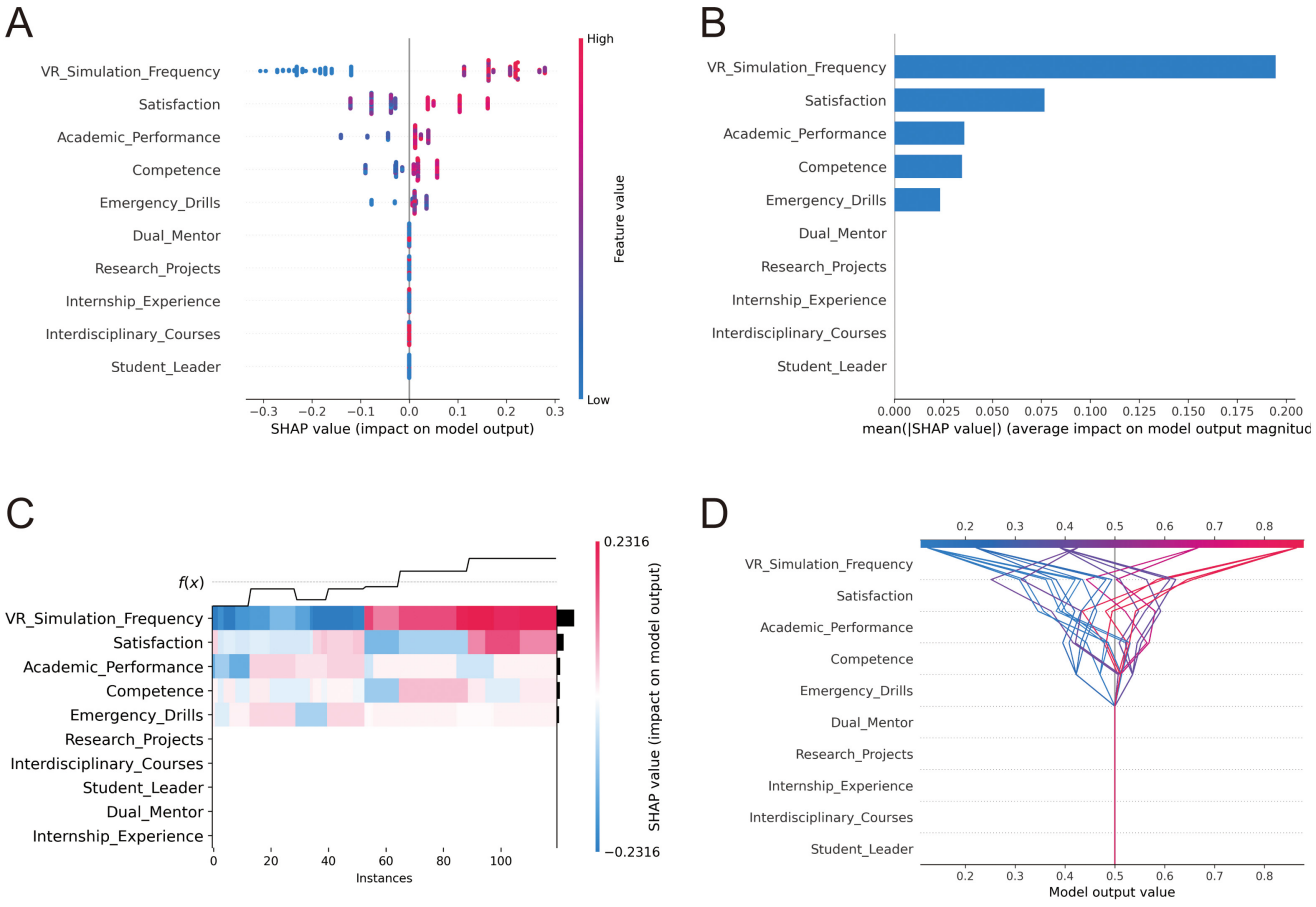
Global SHAP-based interpretation of the final DT model. **(A)** SHAP summary plot showing the direction and magnitude of each feature's contribution to model output, with dot color indicating feature value magnitude. **(B)** SHAP bar plot ranking feature importance according to mean absolute SHAP values. **(C)** SHAP heatmap showing the distribution of SHAP values across individuals and features. **(D)** SHAP decision plot illustrating the cumulative contribution of key features to model output across samples.

To improve the interpretability of the final DT model, SHAP was used to examine the contribution of individual features to model predictions at both the global and local levels. The global SHAP summary plot and feature importance bar plot showed that VR simulation frequency, research projects, academic performance, dual mentor, and emergency drills were among the most influential features in the model (Figures 4A, B). The SHAP heatmap and decision plot further illustrated heterogeneity in feature contributions across individuals and the cumulative effects of key variables on model output (Figures 4C, D).

**Figure 5:**
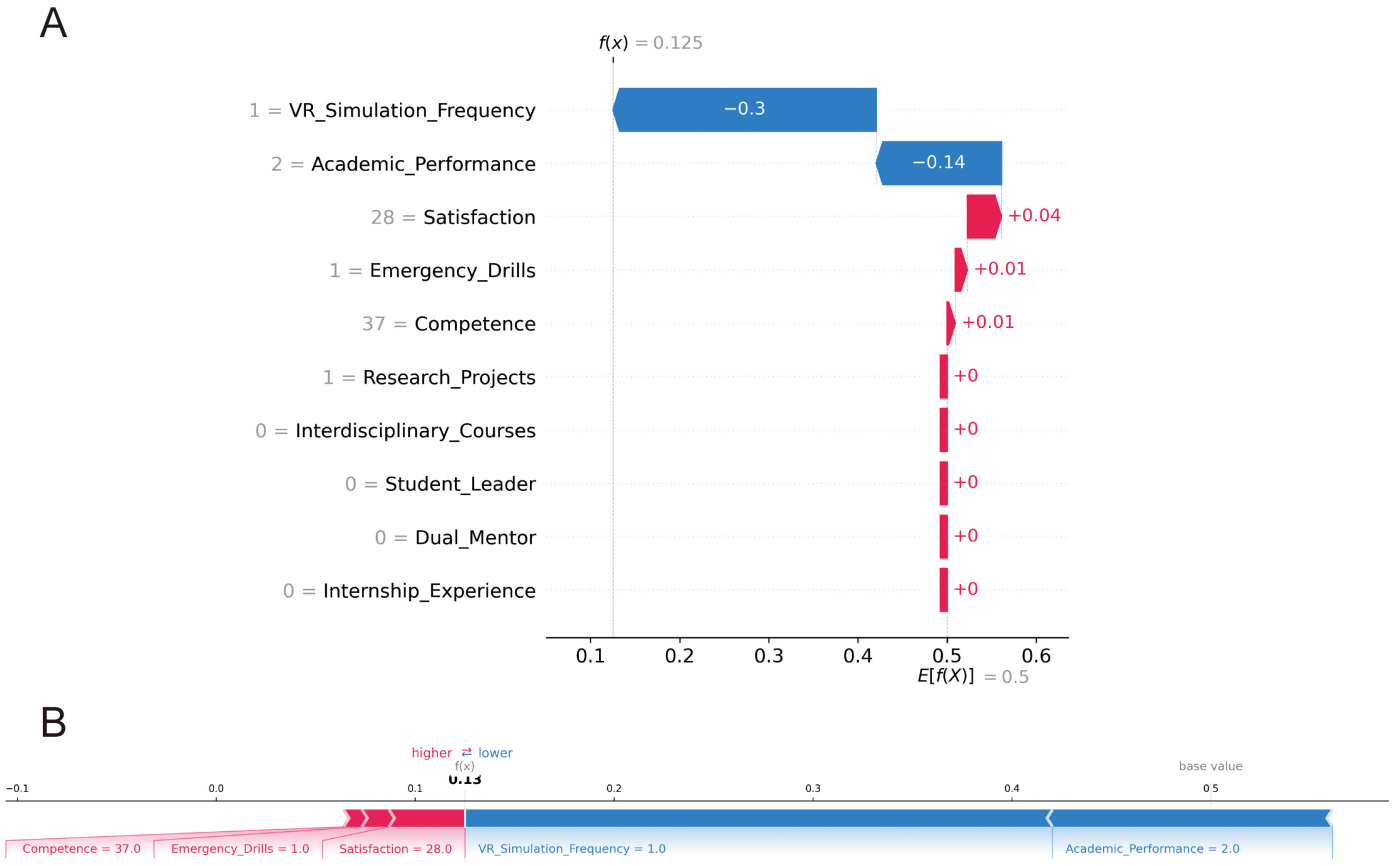
Local SHAP-based interpretation for a random sample. **(A)** SHAP waterfall plot showing how individual feature values contribute to the model output relative to the baseline value. Red indicates a positive contribution and blue indicates a negative contribution. **(B)** SHAP bar plot showing the magnitude and direction of feature contributions for the same sample.

For local interpretation, one random sample was examined using a SHAP waterfall plot and an individual feature contribution plot. We acknowledge that it is merely illustrative and not statistically representative of all students. For this sample, VR simulation frequency (SHAP = −0.21), academic performance (SHAP = −0.14), research projects (SHAP = −0.03), and dual mentor (SHAP = −0.01) contributed negatively to the model output, whereas emergency drills (SHAP = +0.01) contributed positively (Figures 5A, B). These findings illustrate how different educational factors may contribute in different directions and with different magnitudes in individual-level predictions.

## 4 Discussion

This study evaluated the association between an interdisciplinary, integrative, and collaborative curriculum reform and emergency preparedness-related knowledge acquisition among preventive medicine undergraduates. Compared with the pre-reform group, students in the post-reform group showed higher self-perceived competence, greater satisfaction with educational resources, and better objective knowledge performance. In the multivariable regression analysis, interdisciplinary courses, VR simulation frequency, and internship experience were positively associated with the number of correct answers, whereas research project participation showed a negative association. In the supplementary machine learning analysis, DT showed the best overall performance, and SHAP-based interpretation further identified VR simulation frequency, emergency drills, research projects, academic performance, and dual mentor as influential features in the model.

Overall, these findings suggest that the curriculum reform was associated with broader exposure to interdisciplinary and practice-oriented learning experiences, together with better performance across several educational outcomes. This pattern is consistent with the goals of contemporary public health education reform, which emphasize that emergency preparedness requires not only theoretical knowledge but also the ability to apply knowledge in complex and dynamic situations (9, 16, 17), although the present study measured only knowledge-based and self-perceived outcomes.

One of the most notable findings was the role of interdisciplinary courses and VR simulation frequency. These factors were associated with better objective knowledge performance in both the descriptive and regression analyses, and VR simulation frequency also ranked highly in the SHAP-based model interpretation. This finding is educationally plausible. Interdisciplinary coursework may help students connect epidemiology, clinical medicine, environmental health, and health management in a more integrated understanding of public health emergencies (18). Similarly, virtual simulation may provide repeated exposure to realistic scenarios in a safe and controllable environment, allowing students to practice decision-making and apply theoretical knowledge to simulated emergency situations (19). Although the present study does not establish causality, the consistency of these findings across multiple analytic approaches supports the value of these components within emergency-oriented public health training.

Internship experience was associated with better objective knowledge performance in the regression analyses, and emergency drills also ranked highly in the SHAP-based model interpretation. These findings underscore the importance of experiential learning in preventive medicine education. Emergency drills may help students translate abstract concepts into operational understanding, while internships may provide authentic opportunities to observe or participate in real-world public health work (20). For undergraduates preparing for future roles in disease surveillance, risk communication, emergency coordination, and field response, such practice-based components may strengthen the connection between theoretical instruction and professional application (21).

An interesting finding was the negative association between research project participation and objective knowledge performance. This result should be interpreted cautiously. It does not necessarily indicate that research training is unhelpful. A more likely explanation is that the research activities available to students may be more closely related to literature review, methodology, or topic-specific academic tasks than to the content captured by the objective knowledge test in this study. Another possibility is that students involved in research projects may have distributed their time and effort across multiple academic tasks, which could have affected performance on this specific outcome. This finding therefore suggests not that research training should be reduced, but that it may need to be more closely aligned with the goals of emergency preparedness education. For example, student research activities could be better integrated with applied public health problems, field investigation, or emergency-related case analysis.

The machine learning analysis should therefore be viewed as a supplementary approach rather than the primary source of evidence (22). Among the four tested models, Random Forest showed the best overall performance, but predictive ability remained moderate, particularly in the test set. Accordingly, the value of the machine learning analysis in this study lies less in strong prediction than in providing an additional perspective on the relative importance of educational factors and supporting interpretation of the reform components most closely related to student outcomes.

## 5 Limitations

Several limitations should be acknowledged. First, the institution has substantial resources: a national first-class course, a provincial key laboratory, and well-established partnerships with regional CDCs and hospitals. The reform was implemented under the national “New Medical Sciences” and “Healthy China 2030” initiatives. Institutions with different resource levels, student demographics, or policy environments may not achieve identical results. Future studies should consider multicenter designs, longitudinal follow-up, more comprehensive outcome assessment, and external validation of predictive models. Second, the cross-sectional comparison between pre-reform and post-reform cohorts does not allow causal inference, and unmeasured differences between cohorts may have influenced the results. Third, some variables, particularly competence and satisfaction, were based on self-report and may be subject to reporting bias. The objective knowledge outcome was based on a limited number of curriculum-based items and may not fully capture the multidimensional nature of emergency preparedness. Fourth, although machine learning and SHAP offered useful supplementary insights, the predictive performance of the models was modest and no external validation was available. Fifth, because group assignment was based on cohort year rather than randomization, cohort effects and period effects may have influenced the results independently of the curriculum reform. Specifically, unmeasured differences in baseline characteristics between cohorts (e.g., academic aptitude, prior experiences) and changes in the broader societal context across calendar years could partially account for the observed group differences (Selection bias and history bias). Finally, it is important to note that the observed differences in participation rates partly reflect the introduction of new educational resources (e.g., VR simulation was absent before the reform) rather than differences in student engagement alone. Our comparison therefore evaluates the overall effect of the reform package rather than isolating student-level behavioral change.

Despite these limitations, the present study provides empirical support for an interdisciplinary and practice-oriented approach to public health emergency education. The findings suggest that curriculum reform may be associated with stronger self-perceived competence, greater satisfaction, and better objective knowledge performance among preventive medicine undergraduates. In particular, interdisciplinary courses, virtual simulation, emergency drills, and internship experience appear to be important components that merit continued emphasis in curriculum development. These results may help inform future efforts to optimize emergency preparedness training in preventive medicine and related public health programs.

## 5 Conclusion

In this study, an interdisciplinary, integrative, and collaborative curriculum reform was associated with improved emergency preparedness-related knowledge acquisition among preventive medicine undergraduates. Compared with the pre-reform group, students in the post-reform group reported broader participation in interdisciplinary and practice-based learning activities, together with higher self-perception competence, greater satisfaction, and better objective knowledge performance. Interdisciplinary courses, virtual simulation training, emergency drills, and internship experience were identified as important factors associated with better outcomes. Although further multicenter and longitudinal studies are needed, these findings support the value of integrating interdisciplinary and experiential learning strategies into public health emergency education.

## Data Availability

The raw data supporting the conclusions of this article will be made available by the authors, without undue reservation.
